# Impact of male peer‐led outreach on uptake of HIV testing among male partners of pregnant women in Uganda: a randomized trial

**DOI:** 10.1002/jia2.26440

**Published:** 2025-03-26

**Authors:** Faith Naddunga, Michelle A. Bulterys, Agnes Nakyanzi, Deborah Donnell, Juliet Kyomugisha, Juliet E. Birungi, Paul Ssendiwala, Rogers Nsubuga, Timothy R. Muwonge, Joshua Musinguzi, Sue Peacock, Connie L. Celum, Andrew Mujugira, Monisha Sharma

**Affiliations:** ^1^ Infectious Diseases Institute Makerere University Kampala Uganda; ^2^ Department of Global Health University of Washington Seattle Washington USA; ^3^ Departments of Medicine and Epidemiology University of Washington Seattle Washington USA

**Keywords:** heterosexual men, HIV testing, male peer counsellor, pregnancy, Uganda

## Abstract

**Introduction:**

Male partner HIV testing and engagement in antenatal care (ANC) is associated with improved clinical outcomes for men, pregnant women and infants. However, testing rates remain low among male partners of pregnant women receiving ANC in Africa. We evaluated the impact of male peer outreach to increase HIV testing among partners of pregnant women in Uganda.

**Methods:**

We conducted a randomized trial in Kampala, Uganda, enrolling an equal number of pregnant women with and without HIV from public ANC clinics who were randomized 1:1 to intervention or standard‐of‐care (SOC) with delayed intervention after 1 month. (ClinicalTrials.gov ID, NCT05388084). The intervention consisted of male peer counsellors calling male partners of consenting pregnant women and inviting them to test for HIV. In the SOC, pregnant women received an invitation letter to deliver to their partners for fast‐track HIV testing, per national guidelines. We conducted an intention‐to‐treat analysis using modified Poisson regression, comparing the proportion of male partners tested for HIV by month 1 across arms overall and by female's HIV status. A secondary analysis compared the proportion tested for HIV by 3 months after both arms received the intervention.

**Results:**

Between May 2022 and March 2023, we enrolled 150 pregnant women (76 in intervention, 74 in SOC). At 1 month, 18% more males in the intervention arm tested for HIV compared to SOC (32% vs. 14%; risk difference [RD] = 0.18; 95% confidence interval [CI]: 0.05–0.31). This association remained significant after stratifying by female HIV status. HIV testing was 22% higher among male partners of HIV‐negative women in the intervention arm compared to SOC (46% vs. 24%; RD = 0.22; 95% CI: 0.004–0.430) and 15% higher among partners of pregnant women with HIV (18% vs. 3%; RD = 0.15; 95% CI: 0.02–0.28). At 3 months, 50% (38/76) of male partners tested in the intervention versus 35% (26/74) in the SOC/delayed intervention (RD = 0.15; 95% CI: −0.01 to 0.31).

**Conclusions:**

Male peer outreach is a promising intervention to increase knowledge of HIV status among partners of pregnant women. Additional support is needed to increase HIV testing among partners of women with HIV.

## 1 INTRODUCTION

HIV testing for male partners of pregnant women attending antenatal care (ANC) clinics provides health benefits for men, women and their infants [1]. In sub‐Saharan Africa (SSA), pregnancy is a potential entry point for male partners into the HIV care continuum due to high fertility rates and high attendance of ANC services [2, 3]. However, men's HIV testing uptake remains suboptimal; ∼50% of men with HIV do not know their status [4]. Men present for care at more advanced disease stages than women, resulting in worse clinical outcomes [5]. Aside from health benefits to men, male HIV testing and linkage to care can also prevent HIV acquisition in their pregnant partners and infants. Pregnant women have a high risk of HIV acquisition, and vertical HIV transmission is 2–15 times higher among pregnant women with acute versus chronic HIV infection [6]. Among pregnant women living with HIV (PWLHIV), men's HIV testing and disclosure is associated with women's increased retention in prevention of mother‐to‐child transmission programmes [7, 8, 9, 10]. Male partners of PWLHIV may also be at high risk of having or acquiring HIV due to having a partner with HIV [11, 12]. Despite strategies to increase male partner clinic attendance and HIV testing, men's testing uptake remains suboptimal [13]. Men cite barriers to clinic attendance, including travel distance, costs, wait times, confidentiality concerns, stigma, cultural norms and the perception that clinics are places for women and children [14, 15, 16, 17, 18, 19].

To overcome barriers associated with clinic‐based HIV testing, secondary distribution of oral HIV self‐testing (HIVST‐SD) kits from pregnant women attending ANC to give to their male partners has been nationally scaled up in Uganda and across SSA [3]. Studies show that HIVST‐SD can increase HIV testing of male partners of HIV‐negative pregnant women [20]. However, results are based on self‐report by females. Additionally, this strategy places the burden of delivering and explaining HIVST use on females, which is hindered by societal gender norms [21]. Qualitative studies show that both pregnant women and male partners prefer male peer health workers contact men directly to offer HIV testing instead of secondary distribution [22]. PWLHIV, in particular, are reluctant to give HIVST kits to their partners, fearing inadvertent status disclosure, relationship dissolution or intimate partner violence (IPV) [23, 24, 25]. In a discrete choice experiment in Uganda, men preferred HIVST distribution from male peers and had a negative preference for home HIVST distribution from their female partners [26]. In this study, we evaluated the impact of male peer outreach to increase HIV testing among male partners of pregnant women.

## 2 METHODS

### 2.1 Study design

Okutuuka (“*Reach*” in Luganda) was an open‐label, randomized trial of pregnant women attending ANC in Uganda (ClinicalTrials.gov ID, NCT05388084). The primary objective was to evaluate whether a male peer outreach intervention increased partner HIV testing and, secondarily, male uptake of HIV care or prevention (antiretroviral treatment [ART] or pre‐exposure prophylaxis [PrEP]). From May 2022 to March 2023, we recruited women aged ≥18 years and emancipated minors aged 14–17 (i.e. those under the legal age of majority who are pregnant, married, have a child or support themselves) attending a public ANC clinic at Kitebi Health Center III in Kampala. Uganda guidelines permit emancipated minors to consent to research participation. In 2023, 220/11,978 pregnant women tested positive at the Kitebi ANC clinic (HIV prevalence 1.86%). Eligibility criteria were: pregnant, low risk of IPV, having a primary male partner not known to have HIV and who had not tested within the prior 3 months, and willing to provide contact information for partners. Eligibility criteria for men included having a female partner enrolled in Okutuuka and willingness to provide informed consent and undergo HIV testing. Participants were reimbursed 30,000 Ugandan shillings (USD 7.90) per study visit.

### 2.2 Study procedures

We identified 150 women (75 with and 75 without HIV) who were randomized 1:1 to intervention (male peer outreach) or standard‐of‐care (SOC) with delayed intervention using variable size block randomization. At enrolment, women completed demographic and behavioural questionnaires and provided contact information for their partners. Following national guidelines, women in both arms were given an HIVST kit to offer their male partners and an invitation letter for men's fast‐track clinic testing; HIVST became SOC in Uganda in 2018, implemented through directly assisted or unassisted self‐testing [27]. In the intervention arm, a trained male peer counsellor contacted male partners in the following week to invite them for HIV testing and counselling, either at the ANC clinic or in a community‐based location of the partners’ choosing. Male peers were lay counsellors with experience facilitating men's involvement in healthcare in the community, who received training from study staff on study objectives, HIV testing, ART, PrEP, disclosure, and using and interpreting HIVST kits. Peers scheduled time to provide HIV testing and counselling using serial rapid HIV tests and encouraged linkage to ART if males tested positive or PrEP if negative [28]. The peer counsellor continued to contact men over the 3‐month study period until they enrolled or reported not being interested. Women in the SOC arm received the male peer intervention after a 31‐day delay if their partner had not enrolled. Follow‐up visits were scheduled at 1 and 3 months post‐randomization, during which female participants received HIV testing and counselling and completed surveys about recent sexual behaviour and knowledge of their male partners’ HIV testing.

### 2.3 Outcomes

The primary outcome was the proportion of male partners who enrolled and underwent provider‐administered HIV testing by 1 month following female enrolment, both overall and stratified by HIV status. Secondary outcomes were male partner testing at 3 months and men's uptake of HIV care (ART) or prevention (PrEP) depending on HIV test results.

### 2.4 Statistical analysis

Descriptive analyses summarized socio‐demographic information. We conducted an intention‐to‐treat analysis of enrolled women using a binary outcome of male partner testing within 1‐month post‐randomization. Modified Poisson regression models [29] with robust standard errors compared proportions of male partners tested in the intervention versus SOC arms, both overall and stratified by women's HIV status. We computed relative risk (RR) and risk difference (RD) with 95% confidence intervals (CI). In secondary analyses, we evaluated male testing 3 months post‐randomization. Analyses were conducted using R Software (version 9.2). Assuming 15–20% of male partners tested in the SOC arm by 1 month, with equal‐sized randomization groups, we estimated 80% power to detect a 36–42% higher proportion of male partners testing in the intervention arm.

### 2.5 Ethics review

The study was approved by the Infectious Diseases Research Ethics Committee (049/2022), Uganda National Council for Science and Technology (HS2206ES), and the University of Washington (STUDY00014963).

### 2.6 Role of the funding source

The funders had no role in study design, data collection, analysis or dissemination.

## 3 RESULTS

We screened 226 and enrolled 150 pregnant women. Of the 76 ineligible individuals, reasons included having a partner on ART (23), not interested in the study (18) or recently tested for HIV (15). Among enrolled women, approximately half (51%) were living with HIV. Median age was 24 years [IQR: 22–28], 93% were married, 55% were unemployed, 46% had less than primary education and 15% were in polygamous marriages (Table 1). Most women (78%) reported discussing HIV testing with their partner before. Most characteristics were similar by arm and HIV status (Table 1). Among 76 PWLHIV, median time since HIV diagnosis was 2.2 years [IQR: 0.2–5.2] (Table 2). Most PWLHIV were unaware of their male partners’ HIV status; 64% reported their partner had never tested for HIV and 22% reported not knowing. One‐third (32%) of PWLHIV had disclosed their HIV status to their partners, and 33% of undisclosed women reported plans to disclose. No women reported having other sexual partners aside from their primary partner.

**Table 1:** Baseline demographic characteristics: Pregnant women by randomisation arm, by HIV status, and by whether the male partner enrolled within 1‐month post‐randomization

Characteristic	Overall, *N* = 150	Intervention, *N* = 76	SOC, *N* = 74	Pregnant women living with HIV, *N* = 76	Pregnant women not living with HIV, *N* = 74	Male partner enrolled within 1 month, *N* = 34	Male partner did not enroll within 1 month, *N* = 116
Female randomization arm							
Intervention	76 (51%)	76 (100%)	0 (0%)	39 (51%)	37 (50%)	24 (71%)	52 (45%)
SOC	74 (49%)	0 (0%)	74 (100%)	37 (49%)	37 (50%)	10 (29%)	64 (55%)
HIV status at enrollment							
HIV positive	76 (51%)	39 (51%)	37 (50%)	76 (100%)	0 (0%)	8 (24%)	68 (59%)
HIV negative	74 (49%)	37 (49%)	37 (50%)	0 (0%)	74 (100%)	26 (76%)	48 (41%)
Age (years)—median (IQR)	24 (22–28)	24 (22–28)	25 (22–28)	27 (24–30)	22 (20–25)	24 (21–25)	25 (22–28)
18–24	76 (51%)	40 (53%)	36 (49%)	22 (29%)	54 (73%)	22 (65%)	54 (47%)
25–29	48 (32%)	21 (28%)	27 (36%)	29 (38%)	19 (26%)	9 (26%)	39 (34%)
30+	26 (17%)	15 (20%)	11 (15%)	25 (33%)	1 (1.4%)	3 (8.8%)	23 (20%)
<7 years of formal education	69 (46%)	34 (45%)	35 (47%)	46 (61%)	23 (31%)	13 (38%)	56 (48%)
Household had an income every month for the last 3 months	146 (97%)	74 (97%)	72 (97%)	74 (97%)	72 (97%)	34 (100%)	12 (97%)
Unemployed	82 (55%)	43 (57%)	39 (53%)	40 (53%)	42 (57%)	22 (65%)	60 (52%)
Married to partner	139 (93%)	69 (91%)	70 (95%)	68 (89%)	71 (96%)	33 (97%)	106 (91%)
Partner age (years)				32 (27–36)	26 (24–30)		
18–24	23 (16%)	10 (14%)	13 (18%)	5 (6.8%)	18 (25%)	7 (21%)	16 (14%)
25–29	52 (36%)	32 (43%)	20 (28%)	22 (30%)	30 (42%)	15 (45%)	37 (33%)
30+	71 (49%)	32 (43%)	39 (54%)	47 (64%)	24 (33%)	11 (33%)	60 (53%)
Primary partner is the child's biological father	148 (99%)	76 (100%)	72 (97%)	74 (97%)	74 (100%)	34 (100%)	114 (98%)
Lives with partner	127 (85%)	65 (86%)	62 (84%)	59 (78%)	68 (92%)	33 (97%)	94 (81%)
Relationship length (years)	2 (1–4)	2 (1–3)	2 (1–4)	2 (1–4)	2 (1–3)	2.5 (1–4)	2.5 (1–4)
Relationship is polygamous	22 (15%)	12 (16%)	10 (14%)	14 (18%)	8 (11%)	3 (8.8%)	19 (16%)
Has discussed HIV testing with partner before enrolment	117 (78%)	57 (75%)	60 (81%)	51 (67%)	66 (89%)	27 (79%)	90 (78%)
Female delivered HIVST kit to male partner by month 1 follow‐up visit (among women with data)	6/103 (6%)	4/51 (8%)	2/52 (4%)	1/55 (2%)	5/48 (10%)		

Abbreviations: HIVST, HIV self‐testing; SOC, standard of care.

**Table 2:** Baseline HIV/ART characteristics of pregnant women living with HIV

Characteristic	*N* = 76
Time since HIV diagnosis (years)	2.2 (0.2, 5.2)
Has discussed HIV testing with partner before	51 (67%)
Ease of discussing HIV testing with partner	
Very easy	12 (16%)
Somewhat easy	31 (41%)
Neutral	6 (7.9%)
Somewhat difficult	1 (1.3%)
Very difficult	2 (2.6%)
Don't know	1 (1.3%)
N/A	23 (30%)
Partner's reaction to discussing HIV testing	
Very positive	10 (13%)
Somewhat positive	21 (28%)
Neutral	17 (22%)
Somewhat negative	1 (1.3%)
Very negative	1 (1.3%)
N/A	26 (34%)
Has partner ever tested for HIV before?	
Yes, HIVST	1 (1.3%)
Yes, clinic test	7 (9.2%)
No	49 (64%)
Don't know	17 (22%)
N/A	2 (2.6%)
She has heard of HIVST kits before today	24 (32%)
She was offered an HIVST kit at the clinic today	58 (76%)
Has disclosed HIV status to partner prior to enrolment	24 (32%)
Among disclosed women, partner's reaction to learning her HIV status:	
Very positive	13 (54%)
Somewhat positive	7 (29%)
Somewhat negative	4 (17%)
Among undisclosed women, plans to disclose:	
Plan to disclose	17 (33%)
Do not plan to disclose	12 (23%)
Undecided	23 (44%)
Knew she was living with HIV when she got pregnant with this baby	53 (70%)
Tested for HIV during last pregnancy	
Yes	48 (63%)
No	17 (22%)
No previous pregnancy	11 (14%)
Knew she was living with HIV during last pregnancy (among those with a previous pregnancy)	34 (71%)
Received ART during last pregnancy (among those with previous pregnancy and knew she was living with HIV)	33 (94%)
Baby from last pregnancy received ARVs after delivery	
No	7 (20%)
Yes	24 (69%)
N/A (abortion, still birth, infant death)	4 (11%)

Abbreviations: ART, antiretroviral therapy; ARV, antiretrovirals; HIVST, HIV self‐testing kits.

The male peer outreach intervention was associated with an 18% higher proportion of male partners enrolled and HIV‐tested 1‐month post‐randomization than SOC (32% vs. 14%; RD = 0.18; 95% CI: 0.05–0.31; *p*<0.01) (Table 3 ). Most men (89%) tested at the study clinic. The intervention was associated with higher partner testing regardless of female HIV status: 22% higher among partners of HIV‐negative women (intervention: 46% vs. SOC: 24%) and 15% higher among partners of PWLHIV (intervention: 18% vs. SOC: 3%). Across arms, 34 (23%) of males enrolled and tested within 1 month; male partners of PWLHIV were less likely to test for HIV than those of HIV‐negative women (11% vs. 35%; RR = 0.30 [95% CI: 0.15–0.62]; RD = −0.25 [95% CI: −0.38 to −0.12]; *p*<0.01) (Table 3). At 3 months after both arms received the intervention, 43% (64/150) of males HIV tested (intervention: 50% vs. SOC: 35%; RD = 0.15 [95% CI: −0.01 to 0.31]; *p* = 0.06).

**Table 3:** Proportions of male partners who enrolled/tested at the three‐time points post‐randomization, by randomisation arm, and by maternal HIV status, using a modified Poisson regression model

By randomization arm						
	**Intervention** (*N* = 76 women)	**SOC (delayed intervention)** (*N* = 74 women)	**Relative risk [95% CI]**	***p*‐value**	**Risk difference [95% CI]**	***p*‐value**
Men tested for HIV by 1 month	24 (32%)	10 (14%)	2.34 [1.20–4.54]	**0.01**	0.18 [0.05–0.31]	**<0.01**
Men tested for HIV by 3 months	38 (50%)	26 (35%)	1.42 [0.97–2.09]	0.07	0.15 [−0.01 to 0.31]	0.06

**By randomization arm, among women living with HIV**						
	**Intervention** (*N* = 39 women)	**SOC (delayed intervention)** (*N* = 37 women)	**Relative risk [95% CI]**	***p*‐value**	**Risk difference [95% CI]**	***p*‐value**
Men tested for HIV by 1 month	7 (18%)	1 (3%)	6.64 [0.86–51.4]	0.07	0.15 [0.02–0.28]	**0.02**
Men tested for HIV by 3 months	15 (38%)	9 (24%)	1.58 [0.79–3.16]	0.20	0.14 [−0.06 to 0.35]	0.18

**By randomization arm, among women not living with HIV**						
	**Intervention** (*N* = 37 women)	**SOC (delayed intervention)** (*N* = 37 women)	**Relative risk [95% CI]**	***p*‐value**	**Risk difference [95% CI]**	***p*‐value**
Men tested for HIV by 1 month	17 (46%)	9 (24%)	1.89 [0.97–3.68]	0.06	0.22 [0.004–0.43]	**0.046**
Men tested for HIV by 3 months	23 (62%)	17 (46%)	1.35 [0.88–2.08]	0.17	0.16 [−0.06 to 0.39]	0.16

**By maternal HIV status**						
	**Women living with HIV** (*N* = 76 women)	**HIV‐negative women** (*N* = 74 women)	**Relative risk [95% CI]**	***p*‐value**	**Risk difference [95% CI]**	***p*‐value**
Men tested for HIV by 1 month	8 (11%)	26 (35%)	0.30 [0.15–0.62]	**<0.01**	−0.25 [−0.38, −0.12]	**<0.01**
Men tested for HIV by 3 months	24 (32%)	40 (54%)	0.58 [0.40–0.87]	**<0.01**	−0.22 [−0.40, −0.07]	**<0.01**

Abbreviation: SOC, standard of care.

Bold values represent statistically significant *p*‐values (*p* < 0.05).

Of the 34 men tested and enrolled within 1 month, median age was 27.5 years [IQR: 25.0–33.8], and 100% were married and employed. Two men (6%) tested positive for HIV (both in the intervention arm); one newly tested positive, and one was previously diagnosed but not on ART; both linked to ART at enrolment. Of the 32 men tested HIV negative, 19% reported knowing that their female partner had HIV. All HIV‐negative men were offered PrEP, but only one accepted. Most men either declined (78%) or requested time to think about it (13%) (*data not shown*). Women whose partners HIV tested by 1 month were more likely to be married, living together, monogamous, and have lower levels of education and employment compared to women whose partners did not enroll (Table 1). By 1 month, 6% of females reported distributing an HIVST to their male partners, which was similar in both the intervention and SOC arms; HIV‐negative women were more likely than PWLHIV to report HIVST distribution (10% vs. 2%, respectively). After the SOC arm received the delayed intervention between months 1–3 and men in both arms continued to receive calls from male peers, the overall proportion of men who HIV tested increased from 23% to 43%. Participant retention at 1 and 3 months was 73% and 80% among women and 62% and 80% among men, respectively, with no differences observed across arms.

## 4 DISCUSSION

Our male peer outreach intervention offering HIV testing to male partners of pregnant women was associated with an 18% higher likelihood of male HIV testing by 1‐month post‐randomization, with higher rates of HIV testing among partners of both HIV‐negative women and PWLHIV. However, the intervention was more successful in testing male partners of HIV‐negative women than those of PWLHIV. Qualitative studies find that PWLHIV report concerns about relationship dissolution after HIV testing due to inadvertent status disclosure, and both pregnant women and male partners express a lack of confidence that ART adherence prevents transmission (i.e. U = U) [22, 24, 26]. Future interventions focusing on U = U messaging and index partner HIVST may further increase testing coverage [30]. PrEP uptake among HIV‐negative men was low, despite several men reporting that their female partner was living with HIV, which warrants further study.

Barriers to men's facility‐based HIV testing include travel distance, opportunity costs, confidentiality concerns and HIV stigma [14, 15, 16, 17]. Gender norms can affect engagement throughout the HIV cascade: HIV testing, diagnosis and treatment uptake could threaten men's sense of strength and resilience, reputation, self‐esteem, and perceived position as family head [18, 31]. In a qualitative study in South Africa, men viewed HIV testing and treatment to be “reserved for the sick,” and therefore, feeling “too healthy” was a reason to refuse HIV testing and ART [32]. Moreover, societal views of clinics being intended for women and children and not welcoming to male partners discourage men from accompanying their pregnant wives to ANC [33, 34, 35, 36].

HIVST‐SD by ANC clients and newly diagnosed index clients increases partner testing [37], perhaps because African men prefer non‐clinic‐based HIV testing approaches [38]. Previous studies show an increase in male HIV testing after receiving HIVST‐SD from their HIV‐negative female partners; most outcomes are based on women's self‐report [3, 20]. There is a lack of studies evaluating HIVST‐SD among PWLHIV. One trial among 500 women in Ugandan ANCs compared HIVST‐SD to the SOC letter for male partners for fast‐track HIV testing and found no difference between arms; 47.2% of male partners were tested over 12 months [25]. Qualitative studies suggest that men and pregnant women prefer HIVST distribution from male peers over HIVST‐SD from female partners, with PWLHIV, in particular, expressing concerns about adverse events [22]. In this study, we found that the male peer intervention increased HIV testing among both partners of HIV‐negative women and PWLHIV, demonstrating an improvement over SOC HIVST‐SD.

HIVST‐SD can be leveraged outside of ANC. A prior trial of HIVST‐SD to persons with HIV (index clients) in Malawi found that 80% of index clients who were followed up reported offering HIVST to their partners, although only 40% of index clients enrolled were successfully followed up [37]. Another study found offering HIVST to index clients safe and acceptable despite not being associated with identifying more partners with HIV per index than SOC [38]. Finally, one study found distributing HIVST to index clients was associated with higher self‐reported HIV testing among male partners [30].

Our study has several limitations. It was conducted at a single clinic in Kampala, Uganda, with only 3 months follow‐up, limiting generalizability. Second, male HIV testing uptake may be underestimated; some men may have used the HIVST kit that women were provided in the ANC, or tested elsewhere, but did not enrol in the study or share this information with their female partners. Third, we did not collect viral load results for PWLHIV.

## 5 CONCLUSIONS

Male peer outreach to male partners of pregnant women may be a promising intervention to increase HIV testing among male partners of both HIV‐negative women and PWLHIV in Uganda and similar settings.

## COMPETING INTERESTS

The authors declare no conflicts of interest.

## AUTHORS’ CONTRIBUTIONS

AM, CLC, and MS conceptualised the research question. FN, AN, JK, JEB, PS, RN, TRM and JM led the research study and data collection, and AM, CC and MS supervised. MAB and SP analysed the data under the guidance of CLC, AM and MS. FN, MAB, AM, CLC and MS drafted the manuscript with substantial input from all authors. All authors approved the final version.

## FUNDING

The study was supported by the National Institute of Mental Health (C Celum: R01MH113434; M Sharma: K01MH115789).

## Data Availability

Data may be made available by authors AM and CLC upon reasonable request.
